# Hospital Board Infrastructure and Functions: The Role of Governance in Financial Performance

**DOI:** 10.3390/ijerph6030862

**Published:** 2009-02-26

**Authors:** Dan Culica, Elizabeth Prezio

**Affiliations:** 1 Cardiopulmonary Research Science & Technology Institute / 7777 Forest Lane, C – 742, Dallas, TX 75230, U.S.A; 2 University of Texas School of Public Health / 6011 Harry Hines Blvd, V8-110, Dallas, TX 75390, U.S.A.; E-Mail: eaprezio@sbcglobal.net

**Keywords:** Hospital and health system boards, governance, performance, marginal profit

## Abstract

Increased stake of boards in the leadership of the hospitals makes them play a significant role in the financial health of their institutions. Understanding of the correct approach to successfully fulfill this purpose is critical for preparing their organizations for positioning adequately in the health care market. Governmental agencies and public companies, including insurers, will be interested in the extent to which hospital boards have adopted the provisions of accounting reform laws like those introduced by the Sarbanes-Oxley Act. It will remain for the boards to balance their oversight role for financial performance with the pressures of financial accountability.

## 1. Introduction

The fiduciary responsibilities of boards of trustees are better understood than other tasks, but questions still exist regarding the role played by boards in fulfilling specific aspects of these duties. The way boards are interpreting their role in financial oversight and the way they use financial information to make decisions that impact the hospital financial performance are less explored in the scientific literature. The goal of this investigation was to identify whether boards of trustees that proactively adopt theoretical and normative guidelines for the financial oversight process are more likely to achieve better financial performance for their hospitals.

An effective hospital board been shown to be related to high hospital financial performance [1]. The board of trustees has six core financial responsibilities: (1) to specify financial objectives, (2) to review and align the management financial plan with stated objectives, (3) to enhance creditworthiness, (4) to ensure capital is effectively allocated, (5) to monitor financial performance, and (6) to verify financial statements [2]. In a survey of nonfederal community hospitals both Chief Executive Officers (CEOs) and trustees indicated that financial aspects of organizational performance received the most attention during board meetings [3]. Financial performance was the criteria most commonly used to evaluate hospitals and CEOs of hospitals [4,5]. The specific aim of this investigation was to further explore the type of information hospital boards used for financial decision-making and the extent to which the interpretation of financial data related to hospital financial performance. The results will help define activities that boards need to adopt in order to establish the financial oversight necessary to improve financial performance. Such conclusions could be used by the boards of trustees for review and implementation as they adopt principles of evidence-based decision making.

One of the three pillars of hospital governance is *‘overseeing the operations of the organization and the board’* [1,3,5,6]. The infrastructure that boards need to perform effective financial oversight includes appropriate committees and access to expeditiously reviewed information [7]. Relevant information tools available to enable fast decision making are the ‘score-card’ [8] and the ‘dashboard’ [9]. The CEOs recommend indicators for inclusion on these instruments which then underwent periodic review at the discretion of the board of trustees. The ‘balanced scorecard’ concept includes four key dimensions of performance: organizational, executive, quality and financial [8]. Each dimension has its own strategy. Some financial indicators that can be found on a scorecard are cash flow, efficiency, charity care, debt structure, or return on investment. A sound financial strategic plan should also be concerned with aspects of profitability, liquidity, creditworthiness, capital structure, and asset activity [8]. The financial ‘dashboard’ includes specific market conditions (operating margin, personnel expense, and supply expense) and benchmarks relevant to them [8]. It is recommended that these indicators be reviewed monthly with a predetermined plan in place to take appropriate action in the event of negative variances [9]. One study found that ‘dashboards’ included comparisons with other hospitals, but they were used mostly for information purposes rather than for performance management [10].

Part of board’s responsibility to assure financial health of the hospital and health care system is to establish strong processes of operational planning and budgeting, and of monitoring and reporting the progress [11]. Boards should be able to understand the main variances in financial performance and identify correction mechanisms for the management. The metrics for financial evaluation should be compared with national benchmarks. In this sense, governance effectiveness depends on the financial literacy of the board, as well as the participation of board members in continuing education [7]. Hospital boards typically have a diverse number of members, many of whom are not financial experts. Hence a board relies on its finance committee to monitor financial performance, oversee budgeting and capital expenditures, and endowment performance. It is recommended that some members of the finance committee have a business background (retired accountants, treasurers, *etc*.) and that trustees be assigned specific tasks for effective governance [12]. A finance committee should ideally perform several tasks with a certain consistency. Each year members of the committee need to develop a work plan to establish financial objectives. Members must also review financial planning, capital projects and financial conditions, including creditworthiness. In addition, the finance committee has the responsibility to evaluate management activity for financial success. The finance committee needs to determine, at least biannually, if the financial plan is aligned with the strategic plan, and quarterly evaluate operational budgets and review quantitative financial metrics (indicators and standards) [13]. As an increased amount of information becomes available to the hospital boards, healthcare organizations are also forced towards greater transparency [14]. Boards should publish financial reports for their stakeholders every trimester and send internal financial reports every month [15].

Most of the governance studies evaluated the board dynamics and decision making in terms of member selection and interaction. Previous research concerned with hospital financial performance in high performing hospitals looked at the structure of the board and the level of engagement among board members, and emphasized the relationship between the board and the hospital leadership team [5]. Empirical investigations have found partial correlation between financial performance and board effectiveness [1] or between governance configuration and hospital performance [2]. While an association was found between high financial performance and board dynamics, it was not clear how one influenced the other [5]. Thus far the relationship between board activities and functions and hospital financial performance has been explored to a lesser extent. The present study investigated whether boards used the theoretical and practical guidelines described above for their financial oversight process and how this process was correlated to the hospital financial performance.

### 1.1. Study Design

In a governance survey conducted in 2005 more than 75% of hospitals reported having a finance/budget committee [3]. The report further showed that financial performance was the criteria most often utilized to evaluate hospital performance. Hospitals routinely reviewed financial statements and budget performance. Among the hospitals that used benchmarks in their evaluation, financial performance had first priority. These hospitals did not routinely share their benchmarks with the community, or with the managed care organizations. Although financial/business acumen was the second skill set sought when hiring new board members, their knowledge about insurance and managed care were viewed as less important. The present study explored the association between specific structural and functional characteristics of the boards and their financial oversight. Activities relevant to this role depicted from the 2005 survey were examined in relationship to hospital financial performance.

### 1.2. Key Measures

Data about the financial performance of the hospitals included in the study was extracted from 2003–2005 American Hospital Association annual survey. The measures of hospital financial performance used in previous studies in relation to governance were cash flow and operating margin [1]. The Solucient 100 Top Hospitals^®^ National Benchmarks for Success study annually examines changing performance levels in U.S. hospitals and objectively identifies 100 benchmark hospitals based on overall performance. Financial measures employed by Solucient to produce these rankings included expense per adjusted discharge, operating profit margin, cash to total debt and tangible assets [16]. The financial indicator selected for this study was the total marginal profit (operating profit margin) to mirror the profitability of the hospital [17]. The operating profit margin was calculated as a proportion of the difference between total annual revenue and total expenses from the total revenue. This approach was used because the indicator was not affected by institutional size and was also employed by the rating agencies. A summary measure was created to reflect the financial performance of the respective hospitals as an indicator of their overall financial health. This measure was obtained by averaging the operating profit margin over the three year period of the evaluation.

Selected domains of the 2005 Governance survey considered indicative of board infrastructure and its financial oversight processes represented the main groups of independent variables. The structural elements and functional characteristics of the board constituted its infrastructure. The information relevant for board structure included in the study were the selection criteria for new board members, the board size, the number and type of committees the boards were instituting in their hospitals, the compensation mechanisms available for board members, and the presence of the Chief Financial Officer (CFO) as a member of the board. The frequency of annual meetings boards were engaged, the tenure board members were serving in their capacities, the length of terms they were serving on the board, and the use of financial criteria for performance evaluation of the CEO served as measures of board characteristics. The indicators for board financial oversight responsibility were based on whether they used benchmarks to evaluate hospital financial performance, the extent to which they shared these benchmarks and with whom, the actual review of financial performance, and the analysis of financial information on a routine basis. A comprehensive presentation of the content of this survey along with the descriptive results of the responses to its questions can be found elsewhere [3]. Dummy variables were constructed for each response to the questions included in this study.

### 1.3. Data Analyses

Responses to the questionnaire were analyzed to identify how the participating hospitals board’s financial oversight, board structure and board characteristics were associated with hospital financial performance. Specifically, paired t-tests were employed in contingency analyses to estimate the association between these dimensions of board infrastructure and dynamics to their hospital financial outcomes. In addition, the extent to which financial performance was influenced by the board activities and infrastructure was explored with multiple linear regression. The purpose was to test if there was a concordance between the dynamics of financial decision-making processes employed by the boards and hospital financial outcomes. Three regression models were developed to test the association between financial performance and each main group of board traits: structure, characteristics, and processes. Significant associations between board responses and financial outcomes found in the separate regression models were included into a final linear regression to identify the most significant factors in board infrastructure and dynamics correlated with hospital financial performance. While in the contingency analyses financial performance was represented by total hospital profit, in the regression analyses the outcome of interest was operating profit margin calculated as a proportion from the total revenue.

## 2. Results and Discussion

It is generally recommended that board members should hold non-tenured positions on the hospital board, and that the number of terms they serve should be limited. Almost 24 percent of board directors and 19 percent of the other board members of the hospitals that participated in the survey did not have limits in the tenure for their appointments on the hospital board. While having no limits in length of term among board directors did not appear to influence the marginal profit of the hospital, this was higher at the hospitals where the rest of board members had a limit in the terms they served on the board. Having limitless numbers of terms as board directors or board members was not associated with the way hospitals perform financially (Table 1). The financial performance was expressed as the average hospital profit accumulated by the hospitals that participated in the survey over a period of three years including the year of the study.

A viewpoint frequently approached in the debate about board effectiveness is whether board members should be compensated for their work as trustee. Boards used incentives such as participation fees or travel reimbursement to encourage regular meeting attendance as well as participation in continuing education. In this study, although compensating board members for their service did not have a significant impact on performance, hospital profit was higher when board members had an annual fee set for their work, or when trustees received a fee for participating in each meeting.

The extent to which boards operated standing committees varied largely between the hospitals. Generally, there were more audit and budget or finance committees, and less governance entities. In spite of this distribution, the profit was larger at hospitals that did not have such committees. Furthermore, having the CFO serving officially on the hospital board did not contribute to a higher financial performance.

Another aspect of good governance discussed in expert forums was the selection criteria used when appointing new board members. Greater importance was given to diversity of board composition and increased expertise in hospital business mostly in the domains of finance and quality of care. Placing heavy emphasis on financial knowledge as nomination criteria for new board members was not associated with higher financial performance, but this lack of association was not significant. However, having such a competence is important as hospital boards routinely used benchmarks or standards to evaluate hospital financial performance. Making use of benchmarks was reflected in generally higher hospital profit for all the financial indicators employed in the assessment (Table 2). In this sense, regular review of hospital market share made by the board was significantly associated with increased financial profit.

Hospital boards, to large extent, used financial information routinely to evaluate the organizational performance. They also used financial benchmarks to compare their performance with other hospitals, and even shared these indicators internally and with the community. A very large proportion of hospital boards informed their executive bodies and the hospital staff about the level of achievement in financial and other organizational indicators. Sharing benchmarks within an organization was associated with higher total profit, although the accomplishment did not reach statistical significance. The proportion of hospitals that did not inform the communities they served about the overall hospital performance was almost four times larger than the hospitals that did. In contrast, their financial performance was slightly higher than the hospitals who shared their benchmarks with their communities. This situation might be found particularly among the for-profit hospitals.

Although the majority of hospitals made a routine review of financial information, this was not associated with higher financial performance. Boards that regularly assessed budget performance and financial statements had significantly lower hospital profits. Remarkably, a small number of hospitals that did not review budget performance and financial statements routinely, had higher total profits. However, this finding may very well be due to chance.

We tested the boards’ role in higher hospital financial performance with a linear regression controlling for the non-profit status of the hospital, and the hospital size measured in terms of number of beds. Given that the frequency of board meetings was another factor taken into account when measuring board effectiveness, our predictor variable for higher performance was the number of times the board met during one financial year. Boards that met less than six times a year had higher marginal profit on average over three years than hospitals whose boards met more than 12 times every year. Meeting between 7–12 times was associated with lower financial performance than having six or less meetings, but still significantly higher than the hospitals whose boards met more than once per month (Table 3).

Although marginal profit was not related to the size of the boards, financial performance was higher at the hospitals where board officers had no limit for their terms of appointment. In contrast, having no term length for appointing the remainder of the board members was significantly associated with negative financial outcomes. The effect was significantly higher when the board used market share as an indicator. Greater profit was associated with routinely revisiting capital planning. It was quite surprising to find that having an audit committee and systematically verifying the financial statements were correlated with low financial performance. Significantly lower marginal profit was found among non-profit hospitals in contrast with for profits and public hospitals. Similar results were found among the hospitals that shared the performance indicators with their community at large.

There was an overall positive association between higher financial performance and hospital board structure and activity. The number of meetings boards had annually, the tenure of trustee officers on their boards, and use of financial indicators by boards as part of their financial oversight was associated with higher marginal profit over a three year period. It would seem that holding a board meeting almost every month or more often was not a good method to increase financial outcomes. A potential explanation for this finding may be that having meetings spaced out allowed for more time to prepare well-informed reports, which included relevant financial indicators. Board members had more time to get the information in advance and prepare for the meetings accordingly.

The finding that officers’ tenure on the board had a positive impact on financial performance might mean that these positions were not occupied by members of the management team (e.g. CFO) who usually did not stay in those positions for any length of time. It was also important to find that financial performance was much lower when boards did not change their regular membership over time. Trustees of these boards may have served in their capacities over the entire three year observation period of the study and this arrangement did not help the financial situation of the hospital. The difference between board directors and board members might have been due to a more proactive attitude of the former towards hospital performance. Board directors or board chairs usually have vested interests in the condition of the hospitals and health care systems they serve. This finding suggested greater involvement of board chairs in hospital overall health in contrast to the other board members.

Another significant element was the finding that assessing the hospital performance based on specific benchmarks, like market share, led to increased profits. The hospitals whose boards were systematically concerned with capital planning also had higher marginal profits. Giving higher consideration to market share and capital planning indicated that these institutions were more aggressive in their business operations. Using generic measures of assessment (e.g. financial performance) did not influence the marginal profit. This finding might be due to the vagueness of the measure.

Hospitals that were less willing to share their organizational benchmarks with the communities they served performed poorly financially. This appeared to be a characteristic of non-profit hospitals that generally had lower marginal profits than their counterparts. This finding might favor the view that for-profit hospitals were more likely to share their performance information with the communities for higher market penetration [18].

It was surprising to note that hospitals whose boards had an audit committee and those who routinely reviewed financial statements did not show increased financial performance. An audit committee focuses on compliance issues, hiring and meeting with the outside auditor, and addressing any financial reporting irregularities. Lack of evidence of higher financial success might be associated with having inadequate audit committees, or low financial literacy among the board members. The latter explanation may be supported by the other finding that financial acumen in nominating new board members was associated with low hospital profit.

In 2002 the Sarbanes-Oxley Act (SOX) was promulgated as a law to regulate accounting reform [19]. SOX was designed to make organizations more accountable to their exposure to financial risks in relation to their transactions. The most relevant domains for hospital financial health included in SOX were the audit committee and its control over the audit company, the issuance of financial statements and the mechanisms for their internal control, and the ethics code for management and executive compensation [20]. One of the specific provisions of SOX directed the creation of a separate audit committee which was mandated to have at least one expert in accounting [21].

The SOX Act triggered a chain of financial initiatives designed for the non profit sector [2]. Although not-for-profit organizations were not required by law to adopt the SOX prerogatives, it was expected that hospitals and healthcare systems would embrace financial accountability and reporting requirements of the act [2]. However, a survey developed by Clark Consulting showed that very few boards considered implementation of SOX [22,23].

The audit committee has to fulfill certain standards to comply with SOX. This entity centralizes the audit function which creates greater accountability for boards. An audit firm is hired by an audit committee and it should report directly to the audit committee. Members of the committee should all be part of the board of directors, and the overlap of members in the audit, financial and investment committees should be reduced to minimum. Respecting these standards assure that boards have access to findings, and allows them to focus on their oversight responsibilities, holding the management accountable for the results at the same time.

One of the requirements of SOX is for boards to have at least one financial expert in the composition of the audit committee. Such representation is required so as to provide greater credibility due to a better understanding of the auditing process. This improvement in understanding of the auditing process is expected to increase boards’ accountability. Financial statements have to be certified by the CEO and the CFO and then included in the annual audit report. These financial statements report the financial condition of the company, state whether the CEO and CFO established internal controls, describes deficiencies in internal controls, and delineates the corrective actions to be taken to address potential problems. As one of the most important sections of SOX, issuance of financial statements should follow certain guidelines in terms of the time they are released, their content, and their dissemination. Ideally the financial statements should be reported quarterly. Such a timeframe represents a good guarantee for the creditworthiness of the institution and also has the potential to assist the hospital to make a better budget planning. However, it is important that financial statements are supported by proof of internal control mechanisms in relation to financial reporting.

## 3. Conclusions

One limitation of the study was the potential issue of reverse causality (i.e. the governance variables may themselves be affected by the hospital performance). Hospitals in better financial shape may require only few meetings per year, and better financial performance may enable a hospital to acquire greater market share. Similarly, the reviews of financial performance (i.e. use of financial statements) might be the result of poor hospital financial status (as reflected by the negative relationship found in the regression analysis). This issue was a concern because the governance survey was from 2005 and the financial performance data was covering the 2003–2005 period. Consequently, the findings may be able to determine an association between such variables rather than identify a causal relationship.

In summary, boards should meet less frequently and allow enough time between their meetings to accumulate critical indicators of performance for review. Highest preference should be given to have a meeting every other month. The findings of this study reinforced the idea that board membership should be non-tenured, and that board positions should have a limited number of terms for each member. A greater turnover of board members (as opposed to life-terms) would create an increased sense of responsibility towards the community. This could also lead to higher accountability among the boards, and implicitly, the hospitals. It was not clear if compensating trustees for their service on the board helped to achieve better financial performance, although in a very small number of situations there was an association with higher hospital profit. Board compensation might be a function of hospitals with higher profit, which allows them the financial flexibility to reimburse their trustees. Continuing to evaluate hospital performance by using well defined financial indicators (like market share) and defining normative targets (e.g. capital planning) will facilitate the achievement of positive results. Multiple financial benchmarks and their comparisons with nationwide corresponding data may yield even more improvement for the financial health of the hospitals.

Positive associations between board infrastructure and dynamics with higher financial performance found in this study appeared to support the theoretical view of corporate type of boards [17] that place emphasis on fewer, more effective meetings, greater interest in market share, and expansion through large investment in capital planning. Based on the same theory of board typology, elements associated with lower financial performance seemed to favor the philanthropic type of boards [18] with members elected for indefinite (lifetime) terms which may have made them less motivated to be actively involved in hospital performance. The control mechanisms used by these types of boards are less intensive, as they are not looking routinely into their financial statements. In addition, these boards might not have had the right structure for the audit committees in terms of appropriately qualified personnel with adequate financial knowledge.

Issuance of the SOX was meant to refocus the concern of the board onto the overall performance of the organization itself. The board needs to initiate organizational performance assessment, and to envision avenues for improvement in the domains of capital and financial performance, revenue position, cost position, market strength and CEO performance evaluation. The question remains whether making boards more accountable for financial transactions in relation to financial risks will leave them less occupied with financial oversight. There is a perception that pressures from SOX may push boards towards getting involved in details related to the financial transactions for risk management topics rather than analyzing financial metrics and fulfilling their oversight role. Consequently, this situation may create a disproportion in the role played by the boards on financial performance, shifting them more towards financial management rather than financial oversight. A specific aspect for future research would be to inquire whether hospital boards of trustees have adopted directives of SOX and the extent to which this adoption interferes with their financial oversight role.

Overall, more detailed insight is necessary into the process of financial oversight preformed by boards of trustees to identify the type of information they have available and the way boards use the information to make decisions that impact the financial performance of the hospitals they lead. Other financial measures that can be considered for future investigations are the total expense per adjusted discharge [15], the ratio of uncompensated care to total expense, and a composite score consisting of total margin, operating margin, expenses per adjusted discharges and total assets.

Governmental agencies and public companies (including insurance providers) will be interested in the extent to which hospital boards have adopted the provisions of the SOX and the relationship between adoption and their overall performance [19]. It is believed that hospitals and health systems will strengthen their governance by voluntary adopting SOX provisions regarding financial reporting [20]. Under the current pressure for better performance and accountability hospitals are facing, it is assumed that their boards could not afford to ignore adoption of these directives [15]. As one recent document underscored, adoption of SOX may be soon seen as a standard for hospital governance effectiveness [15]. Based upon the conclusions of this study, future Governance surveys should be designed to explore the impact of SOX on hospitals and healthcare systems performance. In addition, relevance of governance to medical outcomes, safety, access to care, community standing and overall costs of care should be examined as well.

## Figures and Tables

**Table 1.** The Board Infrastructure and Total Margin of Profit.

Dimension	Yes		No		P
	N	Profit Margin^*^	N	Profit Margin^*^	value
**No Limit Term Length**					
Officers	372	9.9	1,207	9.7	0.82
Board members	299	8.6	1,280	10.1	0.03
**No Limit number of Terms**					
Officers	878	9.8	701	9.8	0.91
Other board members	700	9.7	879	9.9	0.76
**Compensation for board members**					
Set Annual Fee	38	10.6	1,409	9.6	0.56
Per Meeting Fee	142	11.1	1,333	9.5	0.11
Reimbursement for Travel	344	9.7	1,119	9.6	0.85
Conference Reimbursements	1,180	9.7	313	9.9	0.74
**Standing committees**					
Audit Committee	782	8.7	642	10.8	0.0006
Finance/budget Committee	1,098	9.1	367	11.6	0.0003
Governance Committee	509	8.9	903	10.3	0.03
**CFO member**	357	8.9	1,070	10.1	0.08
**Nomination criteria**					
Financial acumen	799	9.4	780	10.2	0.13
Insurance knowledge	42	10.1	1,537	9.8	0.86
Managed care knowledge	22	9.2	1,557	9.8	0.81

* This represents the mean value of the total margin of profit.

**Table 2.** The Total Margin of Profit and Board Financial Activities among Non-Profit Hospitals.

Dimension	Yes		No		P
	N	Profit Margin^*^	N	Profit Margin^*^	value
**Financial performance used in CEO evaluation**	1,335	9.9	95	10.1	0.85
**Use benchmarks**	978	10	534	9.2	0.18
Market share	674	10.5	905	9.3	0.04
Financial performance	1,051	9.9	528	9.6	0.64
**Sharing benchmarks**					
with Board	1,116	10	463	9.3	0.26
with Management	1,105	10	474	9.4	0.30
with staff	997	10.1	582	9.4	0.27
with community	317	9.1	1,262	10	0.20
**Information reviewed routinely**					
Budget performance	1,503	9.7	76	12	0.07
Capital planning	1,426	9.80	153	9.82	0.98
Financial statements	1,504	9.6	75	13.3	0.006
Operating statistics	1,491	9.7	88	11.2	0.24

^*^ This represents the mean value of the total margin of profit.

**Table 3.** Board Role in Hospital Financial Performance.

Variable	Parameter Estimate	Standard Error
Intercept	14.57	2.15
**Less than 6 meetings a year**	2.55*	**1.08**
**Between7–12 meetings**	1.39*	**0.69**
Number of Board Positions	− 0.021	0.06
**Officers have no limit of term length**	2.13*	**0.96**
**Members have no limit of term length**	− 3.52**	**1.06**
**Audit**	− 1.52*	**0.65**
**Market Share**	1.71*	**0.75**
Financial Performance	0.02	0.81
**Community-At-Large**	− 1.47*	**0.78**
**Capital Planning**	3.46**	**1.27**
**Financial Statements**	− 6.01**	**2.24**
**Non-profit status**	− 3.96***	**0.74**
Number of beds staffed	0.002	0.001

* <.05

** <.001

*** <.0001
